# Book Reviews

**Published:** 1817-04

**Authors:** 


### CRITICAL ANALYSIS
### *OF RECENT PUBLICATIONS.*

*Practical Illustrations of Typhus, and other Febrile Diseases.* By JOHN ARMSTRONG, M.D. Longman, 1816. pp. 305.

NOSOLOGY and nomenclature, falsely conceived and erroneously applied, have proved very considerable obstacles to the progress of medical philosophy. We are taught, in some schools, and in some systems of medicine, to regard a denomination as the predicate of an order of disease, which has its several genera and species marked out in in nature and practice with the precision that is observed in the arrangements of inanimate matter.

Other particulars, connected with the direction of medical studies, have had a share in impeding the advancement of medical science. One of these is the solicitude shewn by many authors and teachers to simplify the causes of disease to an extent beyond the warranty of actual observation. A series of morbid phenomena, for example, is not only supposed to justify a distinct generic appellation, but invariably to depend upon, or to be connected necessarily with, precisely the same internal state; and hence the almost innumerable controversies and disputes about proximate causes. Fever, says one pathologist, is a spasm on the extreme vessels; by another its essence is considered to be a disordered action in the vascular system of the brain. It is an inflammation of the abdominal viscera, says a third; it is not inflammation at all, but debility, replies a fourth. Like the disputants on the chamelion's colour, these several sentiments respecting what fever is, and what it is not, are all right, and all wrong.

Our readers must have remarked the frequency with which we have objected to such terms, and to such attempts at simplifying fevers. The attention we paid very early to Dr. Jackson, and very lately to Dr. Dickson, and some other writers, will readily lead them to believe that we have not been a little pleased to find the subject taken up by one who submits to use the old language, but shows, by experience, the fallacy of opinions attached to such terms.

"It is my intention, (says Dr. Armstrong,) in treating of fevers, to adhere, as closely as the nature of the subject will admit, to the consideration of their phenomena and effects; and to endeavour from these, together with the known powers of remedial agents, finally to deduce some special principles of treatment capable of considerable extension.

"The term fever (he continues) has been used in a very comprehensive as well as in a very limited sense; one set of authors having arranged under it almost every disease attended with increased excitement; and another restricted it to those febrile disorders which have been supposed to be independant of all local affection, and on this account called idiopathic. But, as the disorder of the constitution designated Fever seems always the result or the symptom of some antecedent change or changes in the system, howsoever induced, the most extended signification will be preferred in the following pages. Pyrexiæ shall, therefore, be considered as a class in which three orders are comprehended, namely, fevers proceeding from specific contagions, from marsh and similar miasmata, and from topical affections. The first-mentioned order manifestly admits of a subordinate division, since some of its species only affect the same individual once, while he may be affected with the others repeatedly: but still they all have this in common, that they proceed from, and propagate themselves by, specific contagions. The second order is distinguishable as arising from marsh and similar miasmata; and certainly its species do not *necessarily* possess the power of disseminating themselves by any inherent quality, although they may assume various characters and types. The third order comprehends the topical affections accompanied by fever, and obviously differs from the other two in some points, but chiefly in this, that in it the fever can be traced as the mere effect of the local disturbance; whereas, in the two former, the fever cannot be traced, in the first instance, to any single derangement, but rather seems the product of many morbid actions existing at the same time."

Some of our readers may be disposed to think, that the above extract does not justify the observations we have made on the practical nature of Dr. Armstrong's treatise, since a theory of causation seems to be involved in the very onset of his division of fevers into kinds and species. It will be found, however, that these hypothetical (and, most certainly, disputable divisions) do not influence our author's pathological notions; and that, when he comes to treat of the actual constituents of a certain diseased state, he is not cramped in his ideas of the essentials of such disease by any nosological pre-conceptions. Indeed Dr. Armstrong afterwards criticises his own classification, and owns, that while the doctrine of contagion still remains debatable, "it might, perhaps, on the whole, have been quite as satisfactory to have been directed in the classification of fevers, rather by their phenomena than by their known or supposed origin." We may add, that this *certainly* would have been the preferable mode of designation.

The phenomena of the several disordered conditions to which the term typhus is applied, our author conceives capable of three divisions. The first is *simple* typhus, the second *inflammatory*, and the third *congestive*. That there are irregular determinations of blood even in the first kind cannot but be admitted; but these do not amount to actual inflammations, nor even to those partial and local accumulations of blood in different organs and viscera which are necessary to the constitution of the congestive form of the disease. Dr. Armstrong begins with a very accurate description of the simple typhus. This is too long for transcription; and, as it is merely a faithful history of such symptoms as every one will observe for himself who has an opportunity of witnessing fever, we think the historical detail less necessary here to enter into. His account of the

 inflammatory inflammatory typhus is ushered in by the following observations:

"In pleurisy, and similar disorders, the seat of the disease is always local, its effects general, and its nature inflammatory; but some ingenious authors, with Ploucquet and Clutterbuck at their head, seem to me to have proceeded too far in asserting the same thing of what are called idiopathic fevers. As an example in point, typhus undoubtedly sometimes begins and terminates without topical inflammation; and, as inflammation may occur in one or more parts, without ever producing an infectious distemper, with the true characteristics of typhus, it is evident that inflammation is not its inseparable and essential constituent. When, therefore, this peculiar disease and inflammation are combined together, it appears only reasonable to conclude, that the latter may have been produced by cold, or any other common cause of fever operating with the contagion; or that it may have arisen as an effect of the excitement of the heart and arteries, favoured by some predisposition to inflammation in the part affected; as an inflammatory affection of the chest often arises in the measles, though each may exist independent of the other. Some authors have contended, that the inflammation which accompanies the complicated forms of typhus occurs with the fever, or even precedes it; and others, that it merely follows as a consequence of the general excitement. According to my observations, the local inflammation occasionally commences as soon as the fever itself, but generally arises during the stage of excitement; and hence, perhaps, it may be fairly inferred, that, for the most part, it stands in relation of effect, rather than a cause of the fever. If we reflect that more or less visceral congestion attends the first stage, it will not seem improbable that, by a preternatural distension of the vessels, it may have a morbid tendency in some organ or other which may pass into inflammation by the excitement of the second stage. It usually happens, in mere symptomatic fevers, that the inflammation is limited to one part in particular; but this does not so generally obtain in typhus, for the one organ may exhibit by far the strongest evidences of inflammation, some other part will often be affected in a less degree; which surely favours the notion, that the topical disorders are commonly the products of the general excitement."

Simple, clear, and satisfactory as the above pathology must, one should think, appear to every unprejudiced reasoner and faithful observer, the propriety of viewing the accompanying inflammation in fever in any other light than as a cause of such state, has, we know, been not merely questioned, but pertinaciously denied; while others have again derided the notion of primary inflammation, or even of inflammation at all in any case of genuine fever; and it is this, with other reasons, for which we so much admire the writings of Dr. Armstrong, that he has preferred looking at nature, nature, naked as she is, to *denaturalizing* of facts by hypothetical coverings.

For the same reason that we declined entering into the author's detail of simple typhus, we must forego our desire to follow him through his very accurate statement of the several appearances which are assumed in fever, as different parts and organs become more or less inflamed ; suffice it to say, that the young practitioner who shall peruse the treatise may rest satisfied with the fidelity of the history, as far as any history can be faithful of what is ever varying, and, indeed, never in two cases, in all points, actually similar.

The congestive typhus, Dr. Armstrong thinks, is that condition of fever which has received the least attention by medical recorders, "although some allusions to congestive disease may be found in the antient records of physic, especially in those of Hippocrates, who, under the terms *lipyria* and *epiala*, has often glanced at some peculiar forms of fever, in which there seems to have been internal accumulations, without the regular and universal re-action, which designates the *causus* or burning fever."

Now, although we think that our author has been rather too strict in his division of these different forms of typhus, as they may all exist in a combined state in one individual, and may, moreover, pass from one to another without any regularity, yet we, at the same time, conceive the distinction between congestion, inflammation, and regular re-action, to be of great practical moment. It is of the highest importance to recollect, that "the stage of excitement, without the interference of art, often never emerges at all, or only does so very imperfectly ; the energies of the system either being nearly extinguished by the visceral congestions, or so much oppressed, as to be unable to create an universal re-action."

"The open forms of fever, (Dr. A. continues,) in which heat and arterial re-action are equally developed, will be found the least dangerous ; and those of an obscure nature, in which neither heat nor arterial re-action are equally developed, the most perilous and unmanageable. In congestive cases, the local accumulations obstruct, from the beginning, the common series of febrile phenomena, and there is, in consequence, either a total want of morbid heat, or concentration of it, from partial re-actions, in some particular parts of the body, whilst others are considerably beneath the natural temperature. It is the entire absence or the partial presence of excitement, which constitutes the chief external difference between the severest forms of the congestive typhus, as they all coincide in suppressing the functions, or in deranging the structure of some important organ, by an almost stagnant accumulation of blood in some part of the venous system."

Dr.

Dr. Armstrong states, that Dr. Robert Jackson has the merit of having surpassed his predecessors in explaining the nature of the congestive form of fever; and we have often felt regret that this author has expressed himself in figurative language. Hence doctrines have been ridiculed, as obscure and absurd, which, expressed with a little of Currie's simplicity, or of Armstrong's clearness, might have commanded admiration from the same pens that have been employed sometimes in gross and unmanly censure.

Dr. Armstrong conceives that there is a close analogy between the plague and typhus; and he thinks that those writers and practitioners have been much *at fault* who have indiscriminately regarded and treated the former as a disease of debility. He copies, from the Edinburgh Medical Journal, Sir Brooke Faulkener's description of the three species of plague, and expresses his opinion of their general coincidence in type with the congestive, inflammatory, and the simple typhus.

"Passing from the symptoms, we shall find, (says Dr. A.) that the internal lesions, which the plague produces, also tend to confirm us in the opinion, that it is inflammatory or congestive in its worst forms. Procopius informs us, that, on opening the bodies of those who died of the plague, which raged in the reign of Justinian, a great carbuncle was found within; which language implies, that the patients had died of inflammation in some of the viscera. In those who perished at Marseilles, within the first two days of the attack, the French physicians discovered, by dissection, that some of the viscera were in a state of mortification; and Dr. Mead, in tracing a parallel betwixt the small-pox and the plague, attempts to prove, by positive evidence, that death in both is usually caused by mortifications of those parts. Larrey declares that he has opened the bodies of many persons who died of the plague, and generally found the same appearances: namely, the omentum, stomach, and intestines, gangrenous in some places; the liver in a state of congestion; the gall bladder filled with black fetid bile, and the pericardium with a bloody fluid."

The next section of our author is on the treatment of the simple form of typhus, in the very early stages of which he recommends emetics and purgatives. Respecting the former he says, that,

"In the beginning of almost all febrile complaints, of a simple character, emetics will be found very beneficial; though much neglected now-a-days by many practitioners, probably on account of the universal introduction of purgatives."

And, in reference to the latter, he remarks,

"The system is more torpid than natural in the state of oppression, and of this torpor the bowels largely participate, so that purgatives purgatives ought to be given in such doses as to insure their full operation. Nor need any risk be apprehended from three, four, or even more, copious motions during the day; for, instead of weakening the patient, they will renovate his powers, lighten the system of the load which weighs it down, and contribute to restore the circulation to its healthy equilibrium. Sometimes I have known, at this early period, an emetic and a brisk purgative cut short the fever at once; and where this desirable effect has not been produced, they have hardly ever failed to shorten its duration, and lessen its danger."

The warm bath is recommended in this first stage of typhus; "and there may be cases, in very old or debilitated habits, where, in conjunction with mild emetics, purgatives, and the warm bath, small portions of weak wine may be requisite before the second stage takes place; but, in general, diffusible stimulants are quite inadmissable at this period, because they forcibly tend to make the consequent excitement of the second stage much more violent than it otherwise would have been, and might, in that manner, convert a simple into an inflammatory typhus."

When the stage of excitement has come on, to which the first attention of the physician is most usually called, cold affusion is recommended by Dr. Armstrong; but he thinks discredit has been brought upon this practice by the heat of the surface merely having been made an indication of the propriety of its adoption, while the patient is, at the same time, complaining of internal chilliness. Our author thinks, and we deem the supposition well founded, that Dr. Currie has rather simplified too much in his notions of aqueous affusion, by imagining its virtues ascribable to the reduction of temperature, and influence upon the nervous system merely. He suspects, with Dr. Jackson, "that, whatever may be its immediate operation, the permanently good effects of affusion are to be attributed to the changes which it induces in the circulation." He further intimates that Dr. Currie has overlooked some forms of fever in which cold water is either wholly inapplicable or of limited utility. Purgatives are recommended during the stage of excitements; and we were pleased to see, in this part, some strictures introduced on the mechanical notions, if we may so name them, of Dr. Hamilton, in inferring the utility of purgative medicines too much to their merely evacuating the load of matter from the intestines, and disregarding their still more salutary effect of "restoring healthy *secretion*, and removing irregular distributions of blood from the head, liver, and other vital parts." The diet, Dr. A. tells us, should be exceedingly light through the whole stage of excitement, and "milk mixed with two parts of water and a little a little arrow root, milk whey, barley water, thin gruel, or the like, will answer every purpose of sustaining the powers of the system without exciting the circulation." The vulgar objection to milk in fever, he deems ill-founded.

In the state of collapse, little, our author tells us, will, for the most part, be needed, as the powers of nature will generally be adequate to the recovery, assisted by light nutriment. He cautions against the indiscriminate use of evacuants at this time, and asserts that several cases have come to his knowledge in which patients, thus far advanced in typhus, have sunk very rapidly from the repeated operation of a strong cathartic. When it is necessary to unload the bowels, cordials must be moderately used at the same time, to obviate the exhausting tendency of the purgatives.

We shall conclude this abstract of Dr. Armstrong's directions respecting the treatment of "simple typhus," with the following quotation:

"Having expressed myself strongly against the exhibition of wine in the first and second stages, candour requires me to confess that I have often seen it useful in the last, as, indeed, has already been hinted, in speaking of cerebral oppression from loaded bowels, and of apparent diarrhœa, attended with a state of real debility. But, on the first approaches of the stage of collapse, wine should be sparingly administered, and its effects carefully noticed. If it diminish the irritation, and render the skin universally moist and warm, the tongue softer as well as cleaner, the breathing slower, and the pulse less frequent, and fuller than before, the propriety of proceeding in its use is strongly indicated. Whereas, if the irritation become greater, the skin hotter, the tongue drier, the breathing quicker, and the pulse smaller, and more rapid, its further employment is most certainly contra-indicated; though it should not always be abandoned on the first, but sometimes have the chance of a second trial, after the expiration of a few hours."

In our opinion, it is only required to relieve any immediate symptom, and then only in small doses.

"*Treatment of the Inflammatory Typhus.*"

We believe, with Dr. Armstrong, that the fears of debility in febrile states have operated very injuriously towards preventing the free use of the lancet in states of inflammation accompanying genuine fever; nevertheless, it is of moment to determine between the mere sympathetic fever excited by visceral or local inflammation, and that in which the inflammation is only an attendant symptom,—otherwise we may be overtaken with actual and irrecoverable debility. Dr. A. is, in our minds, rather too free in his recommendation of vigorous depletion in inflammatory affections of the viscera accompanying accompanying fever; for, though "the change which a temporary suspension of animation produces is often strikingly beneficial in phlogistic diseases," and though it may be often very proper to induce this syncope in fever, we question whether it be safe to put such rules into the hands of young practitioners, who are almost invariably disposed to carry principles to an extreme. We must, however, do Dr. A. the justice of stating, that he afterwards qualifies his positions in reference to blood-letting by the following admissions. "In fact, I am no advocate for large and repeated abstractions of blood in the inflammatory typhus, but rather trust to one or two well-timed and moderate attempts by the lancet; and then (he adds) I place my chief reliance upon saturating the system with mercury." Bleeding, our author tells us, should be immediately followed by the use of purgative medicines; "and, if to the agency of these two means that of calomel as an alterative be added, we give a summary of what ought *always* to be attempted, not only in the inflammatory typhus, but in all the varieties of acute visceral inflammation." As far as our experience goes, we think the mercurial plan, namely, that of combining calomel with opium after evacuations, is more applicable and efficient in other forms of visceral inflammation than in those which are attendant upon typhus; but we should be disposed to give up a great deal to the experience and judgment of Dr. Armstrong. Our author terminates his observations on the management of inflammatory typhus by the following judicious cautions:

"Before concluding my remarks on the inflammatory typhus, I beg leave to warn the speculative and the inexperienced from rashly concluding that inflammation exists in every instance in which the head, chest, or belly, are seemingly affected, since opinions deduced with precipitation from a few leading symptoms, may often be extremely defective. Practising in a populous district, I have not unfrequently been called to typhus patients in a state of high delirium, with dry burning skin, parched tongue, flushed face, and red eyes, covered with a load of bed-clothes, confined in close heated bed chambers, and allowed the most improper beverage and diet. On freely ventilating the rooms, extinguishing the fires, removing the superfluous coverings, using the tepid affusions, and ordering purgatives and an antiphlogistic regimen, I have seen a most salutary change induced in a short time, which has been rendered permanent by a perseverance in these very simple means. Other patients, again, have come under my care, who have been treated, in the first instance, much in the same way as those just noticed, some of whom were troubled with a cough and oppression of the chest, and some whose bowels had been been neglected, with much uneasiness and some tension of the belly; but, on prescribing a blister for the first mentioned, and a brisk purgative for the last, with the cooling regimen in both, all the disagreeable symptoms have given way, and recovery has speedily followed. None but those who have had opportunities of contrasting the cooling antiphlogistic treatment with the hot and stimulating, can duly appreciate the superiority of the former, or be fully aware what a pleasing amendment may be frequently produced in mismanaged typhus, by the abstraction of heat, noise, and diffusible stimuli, and the substitution of fresh cool air, sub-acid drinks, a spare diet, and remedies which move the bowels, and tend to take off general excitement and local determinations."

Previously to entering upon the treatment of the congestive typhus, Dr. Armstrong introduces some observations on the nature and management of some other inflammatory affections. Dysentery he regards as an inflammation of the villous coat of the intestines, attended by a congestive or inflammatory state of the liver, and a primary and continued affection of the cutaneous functions; and, in the treatment of this disorder, he recommends calomel and opium, with, in chronic cases, "a long perseverance in the sulphureous Harrowgate water." In erysipelas, he imagines the antiphlogistic regimen is often, in the first instance, too sparingly employed; and, in acute visceral inflammations, no time, he says, ought to be lost in having recourse to the lancet, but the young practitioner ought to be on his guard against the effects of those excessive depletions which "some authors coolly talk about." Fifty, or even more, ounces of blood are recommended by these authors to be taken at a time; but, says Dr. A., in the course of my experience, I have occasionally observed, that, where blood has been so very copiously drawn at one time, it either produced a state of collapse, from which the system never rose again, or was succeeded by indications of violent re-action of the heart and arteries, attended by much nervous irritation." Calomel and opium, after bleeding and purges, are, in these cases, highly recommended by Dr. Armstrong. In acute rheumatism, too, our author has found, by repeated trials, "that early and moderate venæsection, first succeeded by purgatives, and next by calomel, opium, and antimony, is far superior to every other plan." That the mercurial mode of treating rheumatic affections is often highly efficacious, no observer of the effects of medicine can ever question; but it does admit of a question, whether we may not, by a too liberal adoption of this plan in rheumatism, induce that state of the organization concerned in the production of this complaint, which renders the malady more easily excited afterwards afterwards by its several causes than would otherwise be the case. In ophthalmia, Dr. Armstrong thinks that depletion has been generally employed with too feeble a hand; and he imagines that those chronic inflammations of the eyes which have their seat in the tunica adnata are more frequently symptomatic of diseases of the brain than has been commonly supposed. Tic doloureux, too, Dr. Armstrong conceives, for the most part, to be a brainular disease.

"It is certain, (he says,) that persons afflicted with this complaint may live many years, and even sometimes have the appearance of good general health; but it will, I believe, be found that they most frequently die suddenly at last from oppression of the brain, which tends to strengthen the opinion here advanced as to the original seat of the disorder."

### *Treatment of Congestive Typhus.*

The particular nicety of discrimination required in the management of congestive typhus, hinges upon the necessity of distinguishing between real and apparent debility, or, perhaps, more properly speaking, between smothered and exhausted strength. Dr. Armstrong very properly compares the condition of the system, under an attack of congestive typhus, to that of apoplexy, where depletions are called for, in order to remove the load under which the body is sinking; whereas, if the Brunonian notions of stimulating, founded on the idea of debility be acted up to before the congestions are removed, the patient sinks under the combined influence of disorder and treatment; an event, which our author imagines has not seldom happened, in consequence of the want of discrimination just referred to. Blood-letting, however, in this, as well as in the inflammatory form of typhus, requires to be had recourse to very early. Indeed, it is only in the first stage that general bleeding is admissible, "with a view of relieving the local congestion and restoring the natural balance of the circulation;" and more or less blood must be taken, according to the effects which the venæsection produces. Subsequently to the bleeding, Dr. Armstrong recommends the use of the tepid bath, when it can be conveniently procured, and he intimates, that the moderns are rather too neglectful of the importance of restoring the functions of the skin in fevers of the congestive kind by means of warm bathing. After bleeding and purging, our author resorts in these cases to his favourite calomel course. He states, that the power which this medicine has in equalizing the circulation is no-where more conspicuously displayed than in diseases of a congestive character: "for a long time (he says), I overlooked

 one one of the principal effects of calomel in congestive fevers, till at last it was forced upon me by patients almost invariably recovering with rapidity when ptyalism was excited." Blisters laid upon the region of the stomach or liver, Dr. Armstrong likewise recommends in congestive typhus, and "in the worst cases, the moderate exhibition of diffusible stimulants is sometimes really necessary, not only to support the vis-vitæ immediately under depletion, but also to contribute, after its employment, to rouse the heart and arteries, that the natural balance of the circulation may be finally restored." We think the following observations on the use of such stimulants highly judicious and important.

"It will sometimes be requisite to administer cordials, either during or after the evacuations, in order to maintain the strength and equalize the circulation. Yet the precautions which have been so frequently repeated concerning them must not be forgotten here; for they ought never to be considered as an essential remedy in fever, but simply as a mean to obviate some of its consequences, to give a temporary tone to the heart and arteries, or to counteract the debilitating effects of necessary evacuations. Among the preparations in our pharmacopœias, one of the best diffusible stimulants is carbonate of ammonia, which may frequently be prescribed with advantage in congestive fevers, when depletion has been premised; its excitement is neither excessive nor long continued, and if given in moderate and repeated doses, it has considerable influence in determining to the surface."

Dr. Armstrong cautions against the continuance of depletory measures in congestive fever, when the state of positive debility has commenced, even should this be so early as the second day of the disease.

"Sometimes the blood becomes black and dissolved in the most malignant cases of congestive typhus, very early in the disorder, so that when it is drawn it never coagulates, but continues a fluid gore in the vessel. Any approaches (says Dr. A.) to this state of the blood, such as inky petechiæ or dark oozings from the mouth and nostrils, with a weak, quick, thready pulse, always proves that the stage of collapse is at hand, and should make the professional attendant pause before he advances a step forward in the treatment. Depletion is then entirely out of the question, and the judicious use of diffusible stimuli, calomel and opium, together with blisters, and a free ventilation, are the only means to which he can prudently resort at such an eventful crisis."

We find no mention of the Peruvian bark and the acids, which we, however, should prefer in this state of things to the mercurial treatment recommended by Dr. Armstrong. Several observations are introduced in this part of the treatise, on the nature and management of other congestive diseases, eases, but our limits oblige us here to take leave of our author.

On the character and general merits of the work, nothing need be added to what we have already advanced; the style in which it is written may be regarded as a model for medical dissertations; and, where the opinions expressed are at variance with others, we do not find any thing illiberal or offensive in the mode in which they are advocated. Dr. Armstrong is always decided, but never dogmatic—"O! si sic omnia."

---

*A Treatise on Tetanus, illustrated by a Number of Cases.* By JOHN MORRISON, M.D. Small 8vo. Newry: printed for Longman and Co. London. Pp. 122.

WE have frequently felt a wish for some systematic treatise on Tetanus. The cases hitherto given have been scattered in various publications; and few of them taken with the premeditated intention of examining the disease in all its various forms. In this number we shall take the opportunity of introducing another valuable article on the same subject, by which it will appear, that the disease is less frequent than formerly in the West-India islands, though in Demerara, the seat of Dr. M.'s practice, tetanic affections are not less common than in other tropical regions. It may not be amiss to give the author's account of the atmosphere of the place.

Demerara is on the coast of South America. "The land is low, flat, and marshy, abounding with swamps, and (with the exception of stripe along the coast and on the banks of the river, in the cultivation of sugar, coffee, and cotton,) is covered with trees of various dimensions, whose roots, for a great part of the year, lie bedded in water.

"The seasons are divided into the wet and dry. These succeed each other at uncertain intervals, and are of different durations in different years. Moist and rainy weather is by far the most usual; yet there are, now and then, weeks or months of almost intolerable drought.

"In 1804, the 'dry season' was so intense and long continued, that the woods immediately adjoining the cultivation took fire—communicated their flames to some of the cane fields, and a considerable part of the country continued in a blaze for several months.

"The thermometer, during the rainy season, is seldom below 76; or, in the dry, above 86. The diseases most prevalent are intermittents; fever, attended with an inordinate secretion of bile; hepatitis; enteritis; rheumatism; dysentery; and, among children, hydrocephalus is a very common complaint. The subject of this treatise is, in that part of the world, (compared with its occurrence currence in Great Britain,) frequently met with. At one time, I thought of offering to the medical world, through the medium of some periodical publication, all the cases of tetanus which had come under my notice, with, perhaps, some partial remarks: again, on considering that the disease cannot be said to have been treated of separately, I was led to suppose, that an Essay, containing the general history of the symptoms, prognosis, diagnosis, and mode of treatment, accompanied with a few remarkable cases, might be more acceptable, particularly to the younger part of the profession. I hope this attempt will occasion some others, capable of throwing more light on the subject, to appropriate a portion of their time to the consideration of a disease so little understood, yet so fatal in its consequences.

"My opinion, with respect to the cold affusion, is the part which I feel most diffidence in submitting to the public, as I know it differs from that entertained by many of the first physicians of the present day. I trust, however, it will be recollected, that experience, and experience alone, has impelled me to adopt it; and in this opinion I am strengthened by that of a medical friend, who resided for sixteen years in the same quarter, and, consequently, whose field for observation was even more extensive than my own."

In viewing the disease systematically, the author makes no other division than *idiopathic* and *symptomatic*, considering the former distinctions of former authors rather as degrees of severity than of distinct forms of disease.

Idiopathic tetanus, he remarks, is uncommon in temperate climates. It is certainly not only uncommon, but, when it does occur, is usually mild.

"The second species (says Dr. Morrison) is occasioned by wounds or bruises—such as are supposed to lacerate the nerves, small tendons, or ligaments. A partial division or lesion of a nerve, is thought to be attended with more danger than the complete separation of this substance; and I once met with an instance in practice, which favoured this supposition: a negro received a very slight cut in the instep, to which supervened symptoms of lock-jaw, which, however, gave way to some trifling treatment, after a transverse incision had been made a little above the original wound. This man came into the hospital with his injury, at a time when there was a person labouring under idiopathic tetanus in the same house, and whom he was frequently in the habit (from motives of curiosity or friendship) of seeing. I am led to this remark, as it has been mentioned somewhere, that one with a wound, looking at another in the disease, becomes very susceptible of it. The parts that first become affected are usually the jaws, neck, back, loins, and abdomen. The muscles of these parts are rigidly contracted, alternating with intervals of relaxation and ease; perhaps, of the involuntary muscles, the heart itself at length becomes affected.

"The attack of tetanus generally appears to be sudden, and without without any premonitory symptom. It is sometimes preceded by lassitude and uneasiness, want of sleep, faintings, and dimness of sight, and, I believe, in almost every instance, by costiveness: there is a peculiar dejection of countenance, from the very commencement of the attack—'a countenance more in sorrow than in anger,' getting, as the disease advances, strongly expressive of the most melancholy distress. This appearance can scarcely be occasioned by the present pain which the patient feels, nor can it arise from a dread of the return of the spasms, as I have frequently noticed this remarkable appearance of countenance, before the spasms could be said to have begun. So strikingly evident is this feature of the complaint, that a practitioner who has seen but a few cases of it, feels his attention more forcibly arrested by it, than by any other."

The remainder of the description contains the same account of the progress of the disease, as is to be met with in most writers.

"Its duration is, for the most part, in an inverse ratio to the suddenness of the attack, after the supposed cause. This observation, of course, holds good in both species of the complaint. Instances occur, where the symptoms set in very gradually, and the disease is protracted for several weeks. I have known a patient die in forty hours, and one case, where the disease was prolonged for twenty days, and, after all, prove fatal. In the generality of instances, however, where tetanus proves mortal, it carries off the patient before the tenth—often before the fifth day: the younger the subject, the more rapid the disease.

"Sometimes a relaxation of all the muscles takes place, eight or ten hours before death, so that the patient will appear to the attendants in a fair way of recovery.

"The interval between the cause and the appearance of the symptoms, when tetanus takes place as a primary disease, is, I believe, very short. In one of the cases annexed, I think it will appear that the disease set in, in less than twenty-four hours after the cause; and, in the generality of instances of this kind, I am of opinion that the cause does not precede some of the symptoms (costiveness for instance) longer than this period. In that species which arises from external injuries, the latent period will be found to vary in different persons: the symptoms seldom set in till the wound or lesion (when it has been of a trivial nature) be nearly well; and it is the common opinion, that a free suppuration secures the person from an attack."

In cases of small wounds, from splinters or similar causes, the symptoms occur from the eighth to the sixteenth day after the accident. Men are said to be most subject to the disease; but Dr. M. suspects this may be imputed to their exposing themselves more frequently to the causes, such as sudden and violent change of temperature, or accidents producing wounds. The author has met with no fair case of relapse. He has not seen seen it after colica pictonum or enteritis, as described by some. In the warmer climates, though the disease is more frequent, it is said to be milder. This remark, we conceive, relates only to the symptomatic tetanus.

The idiopathic form is, in general, supposed to be the mildest in all climates. This is admitted by Dr. M. yet not in the extent required by some writers, as he has seen many instances in which both forms have terminated favourably.

In all diseases, the prognosis appears to us the most important consideration. It is by this that we can distinguish between the effects of our remedies, and the spontaneous operations of nature ;—by this that we can direct the application of those remedies; and, lastly, by this only that the world at large can judge of the comparative merits of different practitioners. We shall, therefore, make no apology for quoting the whole of this passage.

"Dr. Parry thinks the danger may be estimated in proportion to the quickness of the pulse: 'If, in an adult, the pulse, by the fourth or fifth day, does not reach a hundred beats in a minute, I believe the patient almost always recovers. If, on the other hand, the pulse, on the first day, is one hundred and twenty or more in a minute, few instances, I apprehend, will be found in which he will not die.'*

"I can positively affirm, that, in upwards of twenty cases of the disease (in negroes) which came under my care, there was no instance in which the pulse was in any manner so much excited as in those detailed by Dr. Parry. In a case mentioned by Dr. Curtis, in the Medical Transactions, the pulse is said to have been 'regular' —'*if it deviated at all from the pulse of a person in health, it was rather slow than quick, and somewhat fuller than natural.*'†

"I recollect the pulse (in the absence of spasm) at ninety-eight, in a boy who fell a victim to the disease on the third day; but I cannot say that I have ever remarked it higher than this, either in cases which terminated fatally, or in those which recovered; nor does it appear to me, that we can form any prognosis from the state of the pulse: during the spasms it will be found to rise eight

"* Cases of Tetanus and Rabies Contagiosa.

"† This exactly corresponds with the history of several cases which have lately appeared in different periodical publications.— In a case which I attended in Newry, with Dr. Mollan (now one of the physicians to the Dublin General Dispensary), it was particularly remarked that the pulse was little or nothing affected. The patient died.—In another instance, in Newry, of a patient of Mr. Woods, to whom I was called, about two hours before his death, the pulse (in a child of eight years old) was rather slower than usual at that age. Opium, calomel, and the cold affusion had been used. This patient also died."

or ten strokes in a minute, but, in the state of relaxation, it will deviate but little from that of a person in perfect health. When the disease comes on gradually—when, for the first three or four days, the muscles of the jaws are solely affected, and that, perhaps, not in any alarming degree—when the abdomen is not preternaturally hard, or the bowels obstinately costive—when the skin is moist and moderately warm—and, above all, when the patient enjoys sleep, we may (by the means hereafter to be spoken of) entertain strong hopes of an eventual recovery. An increased flow of saliva, where mercury has, or has not, been used, is always to be regarded as favourable; the less the general air of the countenance is changed, the better. On the other hand, when the attack is violent and sudden—when the muscles of the neck, back, and abdomen, are rigidly contracted—when the patient complains of a shooting pain from the sternum towards the spine—when the belly feels hard like a board, and the least pressure thereon produces spasmodic twitchings or contractions of the muscles of the neck, jaws, &c. or when the same effect is brought about by the presentation of any substance (solid or fluid) near the mouth, we have much reason to fear a fatal termination. Spasmodic startings of the muscles set in sometimes early in the disease, and, recurring every eight or ten minutes, are to be regarded as very unfavourable. Though, in forming a general prognosis, we have reason to fear an unhappy termination, when the symptoms set in violently and suddenly, yet I have met with one instance which formed a striking exception to this rule, in which the disease (the symptomatic species) appeared most violently, and almost immediately after the cause, where the patient's head was bent back, so as to make the body in some manner describe the arch of a circle; yet the person (a boy of about eighteen) got perfectly well."

Some remarks follow on the distinction between *tetanus* and *rabies contagiosa*.

On the appearances after death, we regret to say, that our author is much too short, and, as far as we can judge, hasty, in asserting that the organic derangements, sometimes found, are not necessarily connected with death. On this we shall say more when we come to consider the cases adduced.—This division of the work concludes with a short, but judicious, chapter on Trismus Nascentium, a disease frequent, in the West Indies, in children before the ninth day from their birth, and known, in that part of the world, by the common term of *jaw-fall*.

On the important subject of treatment, we feel ourselves obliged to give the author full credit, because the number of cases he has seen, the attention he has paid to the various forms of the disease, and the success of his practice, fairly entitle him to that respect, until any subsequent events may show that he has been deceived. We shall, however, confine ourselves to his own cases, because we suspect that our readers, like ourselves, must be tired of perusing the opinions and authorities of different writers. Dr. M.'s principal reliance is on opium, mercury, and warm bathing, principally the first. After the cessation of the symptoms, he recommends a continued use of wine and bark.

The five first related cases were idiopathic. Three proved fatal; and we know not how to express our regret that none were examined after death. In the unsuccessful cases, opium was not given early in the quantity recommended by the author. In one of them, the cold bath seemed to increase the symptoms, and to accelerate death. In another the tobacco clyster was twice tried ineffectually. It returned soon each time. The patient died on the fifteenth day, under a remission of all the symptoms.

One of the successful cases was in a female. The quantity of opium exhibited was greater than the author ever witnessed. The other was a male negro, to whom, on the second day, three drams of Tinct. Opii were exhibited every two hours, with mercury and the warm bath. The opium was increased, and wine given *ad libitum*. The warm bath discontinued before the symptoms ceased.

Four cases follow of *tetanus traumaticus* or *symptomaticus*. Of these, three recovered. In all three, opium was given very freely and very early, with wine and ardent spirits. In the fatal case, the patient was bled by her own desire, and could not be prevailed upon to take the opium as directed.

Of the cases which terminated unfavourably, two died after the symptoms had subsided—two during a paroxysm. It does not appear that any of them were inspected. The following are the author's remarks on that subject.

"The bodies of those who die in this disease, run rapidly into putrefaction. Few of them have been examined after death; and those that have undergone inspection, tend to throw but little light on the nature or seat of the disease. To say that it has its seat in the brain or spinal marrow, though these, and every other part of the body, are sometimes found, on dissection, apparently free from disease—or that it depends on something immediately connected with the nervous system, is, in other words, acknowledging that, concerning the pathology of the complaint, we are much in the dark. The fact is, that, in several instances of the most careful examination of the bodies of persons who have died in this disease, no morbid appearance whatever could be discovered, either in the head, thorax, or abdomen; and though it is equally as certain, that, in some cases, the vessels within the cranium have been found turgid—one of the ventricles filled with fluid—the heart closely contracted, and containing but a minute portion of

 blood, blood, yet, having, in several instances, found it impossible to trace any such appearance after death, I think we are warranted in supposing that the organic derangements sometimes found are not necessarily connected with the disease."

In answer to this we would only say, that, if nothing has hitherto been discovered, there is a greater necessity of searching for the *causa mortis*. Mr. Howship, as we have before remarked, discovered, in one instance, this contraction of the heart so firm as almost to resist the knife: but this contraction, like the stiffening of the body, was only temporary, especially in a warm climate. We presume the author would have discovered the same in two of his cases, had they been examined immediately after death. We very much recommend an appendix to this work, containing all that is wanting in the present, namely, an accurate account of every appearance after death, dating the period of examination (which should be as early as possible), describing the texture of the blood and changes which it undergoes, as well as the sound parts. We cannot conclude, however, without thanking Dr. M. for the work in its present form.

---

*Medico-Chirurgical Transactions, published by the Medical and Chirurgical Society of London.* Vol. VII. Part II.—Longman and Co. 1816.

MEDICAL.]—*Case of Inflammation of the Muscular Structure of the Heart.* By EDWARD STANLEY, Esq. Assistant-Surgeon to St. Bartholomew's Hospital.

OUR readers will recollect this case in Mr. Field's Report of Diseases in Christ's Hospital. The particulars are here related afresh, with a more minute account of the examination after death. The great peculiarities discovered were an inordinately thick, dense, and heaving skull, without any diploë—a turbid fluid, with coagulated lymph within the pericardium; the parietes of the heart of a dark complexion; the fibres soft; and,

"Upon looking closely to the cut surface exposed in the section of either ventricle, numerous small collections of dark-coloured pus were visible in distinct situations among the muscular fasciculi. Some of these depositions were situated deeply, near to the cavity of the ventricle; while others were more superficial, and had elevated the reflected pericardium from the heart. The muscular fibres of the auricles were also softened in their texture, and loaded with blood, but without any collections of pus between them. All the cavities of the heart were loaded with coagulated blood. The internal lining, valves, and every other part of the organ, exhibited nothing worthy of remark, except the state of general

 turgescence turgescence in the capillary vessels, which had also extended to the lower part of the trachea, bronchia, &c.

"After this report of the appearances met with on dissection, there cannot, I should apprehend, be any hesitation in viewing the case as an example of acute inflammation attacking the muscular structure of the heart, constituting genuine carditis. It must be admitted that this is a disease of exceedingly rare occurrence: for, in the greater number of cases recorded under this denomination, it would rather appear that the inflammatory action had begun in the pericardium, it being commonly confined altogether to this membrane, and but occasionally extending a little way into the substance of the heart beneath. In those few instances where the heart itself has undergone such changes of structure as have been considered to result from inflammation, it is stated that the organ has sometimes become enlarged, and sometimes small and contracted, and that the muscular fibres have generally been of a pale yellow colour, and much softened in their texture, so as readily to allow the finger to pass between them.* In the work of Corvisart, there will be found references to cases from Barrerus, Forestus, and Fontanus, where distinct abscesses have been met with in different parts of the heart. One such instance lately came under my examination, where a considerable abscess had been formed in the parietes of the left ventricle near to the apex, there being opposite to the situation of the matter, ossification in the lining of the ventricle, with surrounding ulceration. I have not, however, been able to meet with any case on record, where, as an immediate effect of acute inflammation, suppuration has extended generally throughout the muscular structure of the organ.

"The case related in this paper will, I think, be regarded as worthy of record, not merely on account of its rarity, but also on account of its peculiar symptoms. We have here presented to us an instance of inflammation attacking the heart, so violent as to pass immediately into suppuration, and at the same time so destructive as to prove fatal in four days from its commencement, and yet of the symptoms which arose, there was not one which appeared directly referable to the affected organ; on the contrary, from their general tendency, the attention of the practitioners was diverted from the central organ of the circulation, the actual seat of disease, to the centre of the nervous system, where there existed no organic derangement."

On the above we cannot help inquiring, why such a case should be called Rheumatism of the Heart, nor remarking, that inflammation which passes immediately to sup-

"* In one of the cases of Carditis mentioned by Bang in his Selecta Diarii Nosoc. Reg. Frideric. Hafniensis, it is stated that the heart was greatly enlarged, especially in its right side. In most of the cases referred to by Corvisart, it is mentioned that the heart was small and contracted. Frequently, however, no notice is taken of the size of the organ."

puratio puration is less painful, than where the adhesive stage continues longer—the commencing of suppuration being often the cessation of pain. The peculiar thickness of the cranium could, we conceive, only arise from a long-continued increased action; in other words, a chronic inflammation. If the whole system was excited by suppuration in so important an organ as the heart, under such a condition of the skull the patient would be likely to feel pain in the head. These remarks, however, are given with due respect to the gentleman to whom we are indebted for so valuable a case, and, we trust, that a difference of opinion in the cause of certain symptoms will not be construed into a disposition to discourage such communications. We should be highly pleased to see a more minute account of the case of abscess near the apex, slightly mentioned above by Mr. Stanley.

*Sketch of the Medical History of the First Battalion of the First Regiment of the Foot Guards, during the Winter of* 1812 *and* 1813. By John Bacot, Esq.

This well-written paper contains a most interesting, but a most melancholy, account of the mortality of a set of brave men, suddenly engaged in actual campaign after being accustomed only to militia duty, and probably many of them recently taken from the fields or the loom. We regret the account is rather historical than medical. The description of diseases and the treatment is much too short; and we cannot help believing, that, had Dr. Jackson's writings been more attended to, the warm-bath would at least have had a trial in some cases of local congestion, if not high action, about the brain, as appears by the nature of the delirium. We offer this only as a suggestion, from what we have seen of military medicine, and from the descriptions we have read of others.

*Microscopic Observations on the Structure of Bone;* by John Howship, Esq.

This is a continuation of Mr. Howship's ingenious inquiries, contained in the former part of this volume of Transactions. The same accuracy pervades the whole, and the same interest is preserved, but, without the plates, every attempt to explain the subject would be ineffectual.

*Observations on the Condition of the Bones in Rickets, with an Account of some Circumstances not before noticed relating to the Processes of Restoration which take place in them.* By Edward Stanley, Esq. Assistant-Surgeon to St. Bartholomew's Hospital.

This is an ingenious little paper, to show that the manner in in which ricketty bones are curved, their structure, and the progress during restoration. The bones in this softer condition are bent according to the weight they support, and the action of the muscles attached to them. Under restoration, that is, when they harden, this induration commences in that part which may be said to form the centre of gravity, or which is most affected by the action of the muscles, which, of course, correspond with the greatest curvature. Mr. Stanley found also, in the original formation of bone, that the earliest and most considerable deposition of phosphate of lime is in those parts in which strength is most required from the above causes. Some of these remarks, the author observes, have already occurred to the French surgeons. There are two passages not perfectly satisfactory to us. The bones are said to have "exhibited an areolated texture." Does this mean that star-like appearance as if the deposition somewhat resembled crystallization? The other passage is as follows.

"I have never (says Mr. S.) noticed any expansion in the articular ends of ricketty bones, as is mentioned by some authors. I should therefore feel inclined to believe, that there has existed only the appearance of such a phenomenon, the ends of the bones having appeared swollen in consequence of the emaciation of the surrounding soft parts."

Now it is certain, that this deformity sometimes continues throughout life, and in persons not emaciated. May we not, therefore, presume, that the two diseases, though confounded under one name, are different?

*Observations on Tetanus;* by DAVID I. H. DICKSON, M.D. F.L.S. Physician to his Majesty's Fleet, and resident Physician at Clifton.

We have been much pleased with the perusal of this paper, not from any success that attended the author's practice, or to be met with in his reports, but from the discrimination in the forms of the disease.

"This disease (says he) is with propriety divided into the acute and mild, or what I think has inappositely been termed the chronic species; and the latter into the symptomatic and idiopathic varieties. A witness of the first, or acute symptomatic form, from wounds in the cases in question, could not fail to be impressed with the wide difference between these two species in a practical point of view; nor with the conviction that those curative measures which have been described as successful in the one, are almost certainly doomed, in the other, to be followed by unqualified disappointment. The examples that came within my knowledge (but of course there may have been others, with which I am unacquainted) were six or seven soldiers under the care of navy surgeons; geons; and they occurred within a few days after the attack of the 8th of January, 1815, and consequently in an inflamed and painful state of the wounds; but, when the great number that suffered on that occasion, the distance which they were afterwards conveyed, (chiefly in open boats,) and their consequent exposure to the cold night air, and to the heavy rain which fell on the afternoon of the 10th, are taken into consideration, it will rather be a matter of surprise that so few instances followed, than that those which did occur proved so severe and so rapidly fatal. In these cases, as in others, the danger was in proportion to the rapidity and violence of the attack; and they terminated fatally, as far as I am informed, within the second, third, or fourth day; indeed, this form I believe generally runs its course within the third day."

This was accurately remarked by Celsus,* and we know not how it is so generally overlooked by modern writers. The case related by Dr. D. proved fatal on the second day; though, during that short period, the patient had taken ℥iij. of tincture of opium. Some very ingenious observations follow on the state of the circulation, as mentioned by Dr. Parry; on which, the author remarks with equal candour and judgment. We give the following extracts in hopes of further information from future observations.

"As the acute form of traumatic tetanus, to which these remarks are chiefly intended to apply, is so uniformly fatal, it is of the greatest consequence to attend to whatever may assist in detecting the disease early, or in warding it off. Richerand observes, that in wounds threatening convulsions and tetanus, a persevering extension of the limbs during sleep often manifests itself before any affection of the lower jaw; and we should naturally pay more attention to any admonition of this kind in punctured or in extensive lacerated wounds, particularly of tendinous or ligamentous parts; and especially in injuries of the feet, hands, knee-joint, back, &c. in which the disease most frequently supervenes.

"Though it sometimes attacks suddenly, yet, in general, a more gradual invasion affords some prelusive indications of danger: such as increase of pain, irritation, and restlessness, nervous twitchings, pain and difficulty in deglutition, or in turning the head, spasms or partial rigidity of some of the voluntary muscles, pain at the scrobiculus cordis, a suppressed or vitiated state of the discharge, &c. Thus Mons. Larrey adduces several instances of tetanus in which the wound was either dry, or afforded only a scanty serous exudation, and where the symptoms were relieved on suppuration being re-established; and Dr. Reid, in the Edinburgh Medical and Surgical Journal for July 1815, remarks, that on removing the dressing, instead of healthy pus, the surface of the wound was covered with a darkish unhealthy looking matter, which he had in two for-

* "Ea sape intra quartum diem tollunt; si hunc evaserunt sine periculo sunt."—*Cels.* lib. iv. cap. 3.

mer mer instances noticed as the forerunner of tetanus. The torpor of the intestines, which precedes and accompanies this disease, is highly deserving of attention. Mr. Abernethy observes, that in four cases where he inquired into the state of the bowels, the evacuations were not like fæces; and he proposes as a question in investigating the cause, what is the state of the bowels between the infliction of the injury and the appearance of this dreadful malady?"

There cannot be a doubt of the propriety of all these inquiries; but, we must be allowed to express our surprise, that the appearance of the fæces, in four different subjects, should have been ascertained for some days before any suspicion of lock-jaw could have occurred. However this may be, if such was really the case seven years ago, it is inexcusable that the question has not by this time been settled. In many of these cases, we cannot help thinking, that the same cause which induces tetanus, induces also the faulty secretion of the bowels, as well as the change in the appearance of their size, and that they both cease together.

"The exhibition of spirits with opium, as recommended by Mr. Grimstone, with a view of creating, and keeping up for a certain period, a strong action in the system, independently of the support it derives from analogy in those instances where wine has been given in large quantities with success, certainly appears from the cases with which he has favoured us to be well worthy of a further trial; and, in what has been termed the chronic form, he has spoken to me with the greatest confidence of its success. Its failure off New Orleans does not argue any thing against it, under less unfavourable circumstances; for there, as in the African above noticed, the dysphagia increased so rapidly as to render it impossible to administer a sufficient quantity to produce or maintain any considerable effect upon the sensorium. Indeed, in those severe cases, where, as M. Larrey observes, 'the patient has, if not a dread of liquids, at least a great aversion to them;' and where the very attempt to swallow ultimately brings on violent convulsions, it is evident we have little or nothing to look for from the administration of remedies by the mouth; whether this state may be anticipated by opium when foreseen sufficiently early, or there is any charge of arresting it, while forming, by the most powerful exhibition of this medicine, which circumstances might authorise, or how far benefit might be expected from a strong watery solution of it, or by powerfully purgative and stimulating remedies, such as turpentine given by enema, are considerations well worthy of every attention."

All this shows the great difficulty in attempting any thing in the acute state, and, consequently, the uncertainty of our remedies. It is, however, a great satisfaction to learn, that the cases of tetanus were so few in the navy during the superintendance of Dr. Dickson, and we trust the cause of this paucity will gradually develope itself. The paper concludes with with a letter from Dr. M'Arthur, containing four cases, related with equal candour and judgment.

The first was an African boy, in whom spontaneous tetanus had occurred. He lived only forty-eight hours, during the first twenty-four of which he had taken seventy grains of calomel, two ounces of mercúrial ointment were rubbed in, the cold affusions applied eight times; several enemas injected, none of which had returned. The warm bath was afterwards substituted, sixty drops of laudanum given every second hour, and as much wine as he could swallow; a blister applied to the abdomen.

"He died (says Dr. M'A.) in a state of extreme misery about forty-eight hours from the time I first saw him. My attention during his life was directed to the abdomen, which I repeatedly pressed during the absence of spasm, without exciting pain or uneasiness. This case was supposed to have originated in cold, as he had not received any injury or hurt. Having obtained permission to open the body, I found the small and large intestines much inflamed, and in many places approaching to a state of gangrene; other abdominal viscera sound. Having observed sufficient to account for his death, I did not prosecute the dissections farther; a circumstance which I now regret."

The next case was, in symptoms, very similar, though the cause might be a wound.

"Jer. Collins, ætat. 30, a seaman belonging to his Majesty's ship Canada, was admitted a patient into this hospital, on the evening of the 1st of June. The jaws were not completelylocked; most painful contractions of the muscles of the neck and back upon every effort at swallowing or speaking, skin perspirable, bowels bound. The first phalanges of the fore and middle fingers of the right hand had been amputated on board, about twelve days ago, and are now nearly cicatrized. The tetanic symptoms appeared on the morning of the 31st May, soon after he had been on deck for a drink of cold water. He has taken on board, under the direction of Mr. Booth, the very able and judicious surgeon of that ship, laudanum, in large doses, both into the stomach and by glysters. Frictions, with stimulating liniments and the warm bath, were also employed.

"Adhibeatur quam primum enema domesticum. Descendat in balneum tepefactum. Habeat submur. hydrarg. gr. viij. sub forma boli secunda quaque hora. June 2d, unable to swallow the smallest quantity of fluid, and every effort to speak induces the most excruciating distress; no sleep.

℞. Decoct. Amyli, ℥ij.

Tinct. opii, ʒij. M. ut fiat enema. Injiciatur.

Died at 12 o'clock."

In this case, marks of inflammation were discovered along

 the the œsophagus and stomach; cœcum and colon almost sphacelated, and a yellow offensive smelling matter found on the villous coat of both; pharynx, neck, and jaw, as well as the lungs, natural and healthy.

The third case was thirteen days after a successful amputation, with a favourable appearance of the stump. On the first alarm, 18 oz. of blood were taken, the warm bath used, and a purge given of jalap and super-tartrate of potass āā ʒſs. The symptoms increased, and spread, the two following days, to the lower extremities. Calomel given very freely as a purge, with an enema, produced a stool; and, on the following morning, the patient had a spontaneous loose stool. On this day [the 3d] ℥xvj. of blood were taken, after which the pulse rose. Calomel and antimony were given frequently, with camphor. On the 4th,

"Spasms of the muscles of the neck and back; more frequent deep-seated pain under the sternum; pulse low; perspires freely; one stool. Repetantur pilulæ ut heri. Adhibeatur emp. lyttæ sterno.

"5th. Much as yesterday, perspires less, bowels bound. Repetantur omnia. Injiceatur enema catharticum.

"6th. Slept better than he has done for several nights; muscles of the jaw more relaxed; spasms much as usual; had an enema last night, which is still retained; stump unhealthy. Continuentur pil. et baln. calid. Injiceatur enema.

"7th. Passed a bad night; spasms severe; a gangrenous spot on the anterior edge of the stump; one free stool. Continuentur omnia ut heri. Habeat etiam haust. anod. ex tinct. opii ʒj. hora somni.

"8th. Every appearance of approaching dissolution; inside of the lips slightly ulcerated, as if by the action of mercury; no ptyalism. Died at 2 p. m.

"During the continuance of the disease, the jaws were not so much shut as to prevent his receiving nourishment, especially after the second bleeding.

"*Dissection.*—Stomach much drawn up under the diaphragm. Intestines in several places inflamed, chiefly the ileum; stomach and intestines inflated with air; no mark of disease in the œsophagus, pharynx, or larynx."

The fourth case followed amputation of the arm at the shoulder-joint, and commenced twelve days after the injury and eleven after the operation, till which time every thing was going on well.

"Dec. 16th, was restless, and somewhat delirious in the night, pulse sunk, granulations of the wound pale, bowels open. His wine and porter to be continued.—Haust. anod. hora somni.

"17th. Complained last night of stiffness of the jaw, and contractions tractions of the muscles of the face and neck; took sixty drops of the tinct. opii at bed-time, and repeated it in the night. This morning the spasms of the neck extended to the back, and are increased in frequency and violence; pulse quick, and scarcely perceptible. Habeat tinct. opii ʒi. tertia quaque hora, vel pro re nata.

"18th. Spasms continued with unremitting violence. Was put into the warm bath last night. Died at 9 o'clock this morning.

"*Dissection.*—Stomach singularly contracted, internal coat exhibiting appearances of inflammation, and much yellow-coloured mucus adhering to it. Portions of the intestines inflamed, particularly the ileum; a very extraordinary yellow fluid, resembling that in the stomach, was in great abundance in every part of the internal canal, which, upon cutting into the intestine, effervesced on coming in contact with the external air; liver diseased; did not examine the thorax or head."

In all these cases, related with the greatest candour, and, doubtless, with equal accuracy, we find the symptoms progressively increasing till death. The examinations, we regret to say, are deficient in the following points:—we are not told how long after death the body was opened, and no mention is made concerning the contraction of the involuntary muscles, particularly the heart. The cases are, however, highly important as far as they go. The author confirms the remark of Dr. Dickson, that tetanus is much less frequent in the West-India fleet than formerly; he adds, indeed, that it is scarcely known.

*Account of a Case of curious Imperfection of Vision.* By WHITLOCK NICHOLL, M.D. of Cowbridge.

The incapacity to distinguish colours is by no means uncommon. In this, as in the instance related in the Philosophical Transactions, the peculiarity is not confined to a single individual in the same family. It has descended, like many others, from the grandfather and his brothers, missing a generation.

*Some Observations on a Species of Pulmonary Consumption very frequent in Great Britain.* By ALEXANDER P. WILSON PHILIP, M.D. F.R.S. Ed. of Worcester.

"To many physicians (says Dr. P.) there will probably be nothing new in the following observations; but this remark, I believe, will not apply to the greater number of medical men, because it is very common for the different species of pulmonary consumption to be regarded as the same disease, and treated in the same way."

This modest distinction between *physicians* and a great number of *medical men*, appears to us ill suited to a society consisting of physicians, surgeons, and esquires. However, for proofs that different species of consumptions have been noticed for nearly twenty years, we appeal to our own Journal; and, we believe we may say more than thirty years, to Dr. Moore's "Travels in Italy," which contain two very long letters on that subject only. We pretend not to say that in either of these publications the particular species described by the author of this paper is noticed. Dr. Philip, however, in referring us to the second edition of his Treatise on Febrile Diseases, published twelve years ago, helps us to an author who, with others, was not entirely inattentive to the liver as a cause of consumption.*

Dr. P. tells us it is not his intention to give a detailed account of the symptoms of this species of phthisis, but only the symptoms and modification of symptoms by which it is distinguished. It may not, however, be amiss to remark, in general, among these *symptoms*, that, in the early stage, the spirits are often depressed, and the countenance sallow; the cough generally dry and severe, with mucus expectoration from the violence of its paroxysms, which occur oftenest after eating, or on lying down, especially on one side; the matter, from being at first limpid or glary, becomes purulent; the pain, if any, is dull at the pit of the stomach; the hectic later in its appearance, and the emaciation slower. Many of these symptoms, and some others mentioned by Dr. P., the reader will perceive are not peculiar to this species of phthisis. Of this the author seems aware.

"Such (says he) is the manner in which the symptoms common to all forms of phthisis are modified in this species of it; but a

---

* We have not Dr. Philip's second edition, but, in the 4th vol. published according to the date he mentions (1804), is the following passage:

"One cause of tubercles, which appears to me to operate very frequently, has been but little noticed by writers—Raulin and some others mention it—obstructions of the abdominal viscera, particularly an enlarged and indurated state of the liver, cases of which I have known prove fatal, by inducing phthisis, without the practitioner having been aware of the cause which supported the disease. The formation of pus seems, in most of these cases, at an early period, to be unattended with ulceration, sometimes, perhaps, even with tubercles, for I have seen many supposed to labour under phthisis, speedily relieved from the most urgent symptoms, by the means which remove the enlargement and induration of the liver. If the case is neglected, however, tubercles and ulceration succeed, and then it is too late to afford permanent relief."—*Dr. Philips Wilson on Febrile Diseases*, vol. iv. p. 518 and 519.

diagnosis diagnosis resting merely on the modification of symptoms must always be fallacious; it is, therefore, fortunate that, in the present instance, there is superadded to the usual symptoms of phthisis, others peculiar to this species, by which, with very little attention, it may always be distinguished; symptoms indicating a deranged state of the digestive organs. The patient is often distressed with flatulence and irregular bowels, the tongue is furred, the appetite for the most part, contrary to what is usual in other forms of the disease, much impaired, the fæces seldom well coloured, and the epigastric region more or less full and tender on pressure. These symptoms vary much at different times, but the patient is hardly ever free from them. The connexion between them and the pulmonary symptoms is rendered evident by the latter increasing with the former, so that when the epigastric region is very full and tender, the cough and dyspnœa are more troublesome; and on the fulness and tenderness subsiding, the pulmonary symptoms abate. Even the rising of the wind from the stomach often, for the time, removes the tendency to cough.

"The foregoing are the symptoms of the more early stages of that species of phthisis which I am endeavouring to distinguish, because I have found it requires a very different plan of treatment from other forms of this disease."

"*Of the Causes.*—The species of phthisis which I am considering arises from all the causes of this disease, with the exception of those whose operation is confined to the lungs themselves, the inhaling of dust, other diseases of the lungs, the bones pressing unequally on them, &c. yet its causes are not less numerous than those of other forms of phthisis. To compensate for the want of those causes immediately affecting the lungs, we have a numerous set of causes affecting the digestive organs. Drunkards, at that time of life which disposes to phthisis, frequently fall a sacrifice to this form of the disease; and those who have been long subject to severe attacks of dyspepsia, and what are called bilious complaints, are liable to it. In short, we perceive equally in its causes, as in its symptoms, its connexion with the state of the digestive organs, from which it may be justly termed dyspeptic phthisis. It particularly deserves attention, that, in many families, this form of the disease alone appears. When this is the case, its fatal effects may generally, I believe, be prevented by carefully avoiding the causes which tend to debilitate the digestive organs, and watching the first approach of the disease.

"*Of the appearances on dissection.*—The appearances of the lungs are generally much the same as in other cases of phthisis; but we almost always find, at the same time, either a diseased state of the liver, or traces of disease having existed in it. In cases where the disease of the liver has been severe, and the patient has died as much of this disease as of that of the lungs, I have *repeatedly* found those parts of the lungs in the neighbourhood of the liver alone affected, the left side appearing sound, or nearly so.

In

In general, however, the affection of the liver seems to have little immediate share in the cause of death; and the patient lives, as in other cases of phthisis, till almost the whole lungs are rendered incapable of their function. Here, as in many other cases, we often have occasion to remark to what extent change of structure, even in the vital organs, may go without destroying life, when the change is very slowly effected; a circumstance which, perhaps, more than any other, shews the resources in the structure of the body itself, against the effects of disease.

"It is not at all uncommon, in dyspeptic phthisis, to find the spleen, as well as the liver, diseased. As the cœliac artery, dividing into three branches, supplies the liver, stomach, and spleen, may we not suppose that the pain so frequently felt in the left side and in the stomach, in this form of phthisis, arises from more than the due quantity of blood being thrown into the vessels of these organs, in consequence of the obstructed state of the liver? is it owing to their being supplied by the same artery, that we so frequently find a diseased state of the liver and spleen in the same subject, and that inflammations of these organs so frequently alternate with each other? I have repeatedly seen the inflammation pass from the one organ to the other, and back again to that first affected."

Having thus traced the *symptoms*, *causes*, and *appearances* on dissection, the author is prepared to inform us of the *nature* of Dyspectic phthysis. Of this we shall offer the first paragraph, as many of our readers will, by that, be enabled to guess the rest.

"It is impossible (says Dr. P.) to observe, even in a cursory manner, the symptoms of this disease, without remarking that the state of the lungs is connected with that of the digestive organs. Its causes we have seen afford the same inference; and, in those who die of it, I have just had occasion to remark, we very frequently find a diseased state, or proofs of a diseased state having existed in one of the organs of digestion, whose intimate sympathy with the others, numberless observations prove. A question of the first importance in the treatment of this disease here arises. What is the nature of the relation observed between the affection of the lungs and that of the digestive organs, in this species of phthisis? is the one a consequence of the other, or are they simultaneous affections arising from a common cause? They are not simultaneous affections, for the one always precedes the other. In by far the majority of cases in which both the lungs and digestive organs are affected, the affection of the digestive organs precedes that of the lungs. In some instances, we find the affection of the lungs the primary disease. But in these, the case does not assume the form above described, but that of simple phthisis; and the hepatic affection, which is always the most prominent feature of this derangement in the digestive organs, does not shew itself till a late late period of the disease, and then little, if at all, influences the essential symptoms."

As may be supposed, Mr. Abernethy is soon unexpectedly met on the road (for who can fail to meet on any of those hacknied roads which require no guide); and, because it is found that mercury will, by exciting a new action, cure many chronic diseases; that most chronic, and all acute, diseases, affect the digestive organs; and, because mercury is supposed a specific in all diseases of the liver, that therefore all chronic diseases arise from a vice in the digestive organs, and that such vice may generally be traced to the liver: hence, that mercury will cure all chronic diseases. It appears, however, that the mode of giving mercury is different from Mr. Abernethy's. Might we not, therefore, ask whether Dr. Hamilton's practice of purgatives, without mercury, would not be equally efficacious? and less dangerous; inasmuch as a number of medical men, (not many physicians,) finding a little mercury so universally efficacious in slight complaints, may think that, in more obstinate complaints, a great deal would be equally so; and thus, by degrees, a whole town, or a certain range of it, may be found in a state of high salivation.

The paper contains also some long remarks concerning sympathy, and some shorter ones concerning scrofula. The following passage surprised us much.

"It always adds much to the unfavourable prognosis, to find that the patient has scrofulous enlargement of the more external glands, which is often such as cannot be seen, but only felt. It will be generally admitted, I believe, that external glandular swellings and suppurations often tend to prevent internal disease. We see, in the same family, some fall a sacrifice to phthisis, while others, labouring under these swellings, escape it. I have seen a person in the last stage of phthisis saved by the glands of the neck suddenly swelling and suppurating. But that slight enlargement of the external glands which may be rather felt than seen, while it indicates, is not of sufficient importance to obviate the tendency to internal disease."

This paper is very incorrectly printed—besides *scrophula* for *scrofula* in the above passage, we read that "animal food and *fomented* liquors are peculiarly injurious."

*Facts illustrating the Effects of the Venereal Disease in the Fœtus in Utero, and the Mode of its Communication.* By WILLIAM HEY, Esq. F.R.S. &c. &c. of Leeds.

Nothing can ever shake the reputation of Mr. Hey's former writings. They are replete with solid judgment, accurate description, and perspicuous reasoning. If, therefore, we object to this paper, it is only to urge *non omnia possumus*

*possumus omnes.* Mr. Hey probably began his useful career long before Mr. Hunter was at all understood, and when the venereal disease was described not as a morbid poison whose character was to be accurately ascertained; but taken for granted according to suspicious circumstances, and the cure by mercury. According to the first, all *local* diseases appearing on certain parts, and oftentimes on any parts, were at that time pronounced venereal; and, according to the second, all, or most, *constitutional* diseases must be venereal, as they are usually attended with dyspepsia, and mercury is with some the universal remedy. Our respect, however, for one who has done so much for surgery, and is still in the full vigour of intellect, prevents our treating the subject with levity. We shall, therefore, only remark, as the local symptoms of his cases are no-where described, it is not for us to oppose what we cannot be acquainted with. To explain ourselves better, we offer the following extracts, with which the paper concludes.

"When the notice of syphiloid diseases was first laid before the public by Mr. Hunter, the chief force of his argumentation was derived from this consideration, that, although the diseases had a considerable resemblance to syphilis, yet being cured without mercury, they were thereby proved to be of a different nature. Now if this reasoning is allowed to have validity, we may suppose the converse of it also to be valid. If the disease in question have the usual symptoms of syphilis, and will yield to no other remedy than mercury, we may fairly conclude that it is syphilitic. What is our design in giving distinct names to diseases, but that we may thereby be directed to select the appropriate remedy for their cure? If my patient obtain the desired benefit from my nomenclature, a defect in my theory may well be excused.

"It may justly excite surprise, that the gentleman whose case I have last related, should have remained free from disease, when his wife was in the condition which I have described. I confess myself unable to account for this circumstance, without calling in the aid of a supposition which wants probability. I have related all that I know of the case, and must leave the solution of the difficulty to your better judgment.

"As I do not mean to enter upon an examination of the distinctions which have of late years been made betwixt genuine syphilis and syphiloid diseases; I trust I shall not be considered as having, by the preceding observations, expressed any doubt of the accuracy of those distinctions. I rejoice to see the ardour and judgment with which every branch of medical science is now cultivated; and must ever rank myself amongst the pupils of those, who by their discoveries and successful labours, are daily diminishing the sufferings of mankind."

The concluding sentence is worthy of such an author, and the the modesty of the whole paragraph might almost disarm criticism. But, where did Mr. Hey collect Mr. Hunter's chief argument, "that, although the diseases had a considerable resemblance to syphilis, yet, being cured without mercury, they were thereby proved to be of a different nature;" and, if such is not Mr. Hunter's chief argument, how much must we regret the logical allusion to the converse of such reasoning. Mr. Hunter objects equally to some cases which required frequent and long repetitions of mercury, as to others which were cured without any; and uses both only among the arguments, that there is "an ambiguity attending these diseases in all their stages—in the mode of their being caught, *the appearance*, and the cure."—*Treatise*, p. 397, Second Edit.

*On the Medicinal Properties of Stramonium; with illustrative Cases.* By ALEXANDER MARCET, M.D. F.R.S. Physician to Guy's Hospital.

It appears, by this paper, that Dr. Marcet, prudently availing himself of a hint from one of his pupils, (a son of Mr. Norwood, of Ashford,) directed extract of stramonium in a very violent case of sciatica. Encouraged by success, he has continued its use in many similar cases, as well as in tic douloureux, and with apparent success. We very much approve of these means of multiplying our resources. It was found by Dr. M. that the most efficacious extract is made from the seeds.

[The Chirurgical papers will be attended to in our next.]

